# FARP‐1 deletion is associated with lack of response to autism treatment by early start denver model in a multiplex family

**DOI:** 10.1002/mgg3.1373

**Published:** 2020-06-25

**Authors:** Francesca Cucinotta, Arianna Ricciardello, Laura Turriziani, Giorgia Calabrese, Marilena Briguglio, Maria Boncoddo, Fabiana Bellomo, Pasquale Tomaiuolo, Silvia Martines, Marianna Bruschetta, Francesca La Fauci Belponer, Tiziana Di Bella, Costanza Colombi, Marco Baccarin, Chiara Picinelli, Paola Castronovo, Carla Lintas, Roberto Sacco, Thomas Biederer, Barbara Kellam, Stephen W. Scherer, Antonio M. Persico

**Affiliations:** ^1^ Interdepartmental Program "Autism 0‐90" "G. Martino" University Hospital of Messina Messina Italy; ^2^ Department of Psychiatry University of Michigan Ann Arbor MI USA; ^3^ Mafalda Luce Center for Pervasive Developmental Disorders Milan Italy; ^4^ Service for Neurodevelopmental Disorders & Laboratory of Molecular Psychiatry and Neurogenetics University “Campus Bio‐Medico” Rome Italy; ^5^ Department of Neurology Yale University School of Medicine New Haven CT USA; ^6^ Genetics & Genome Biology Program Toronto Canada; ^7^ The Centre for Applied Genomics The Hospital for Sick Children Toronto Canada; ^8^ Department of Molecular Genetics University of Toronto Toronto Canada; ^9^ McLaughlin Centre University of Toronto Toronto Canada

**Keywords:** autism spectrum disorder, biomarkers, FARP1, neuronal plasticity, treatment outcome

## Abstract

**Background:**

Children with autism spectrum disorder (ASD) display impressive clinical heterogeneity, also involving treatment response. Genetic variants can contribute to explain this large interindividual phenotypic variability.

**Methods:**

Array‐CGH (a‐CGH) and whole genome sequencing (WGS) were performed on a multiplex family with two small children diagnosed with ASD at 17 and 18 months of age. Both brothers received the same naturalistic intervention for one year according to the Early Start Denver Model (ESDM), applied by the same therapists, yielding dramatically different treatment outcomes.

**Results:**

The older sibling came out of the autism spectrum, while the younger sibling displayed very little, in any, improvement. This boy was subsequently treated applying a structured Early Intensive Behavioral Intervention paired with Augmentative Alternative Communication, which yielded a partial response within another year. The ESDM nonresponsive child carries a novel maternally inherited 65 Kb deletion at chr. 13q32.2 spanning *FARP1*. Farp1 is a synaptic scaffolding protein, which plays a significant role in neural plasticity.

**Conclusion:**

These results represent a paradigmatic example of the heuristic potential of genetic markers in predicting treatment response and possibly in supporting the targeted prescription of specific early intervention approaches.

## Graphical abstract

This is the first study addressing the involvement of *FARP1* in a human disorder and the genetic basis of response to early behavioral intervention in child psychiatry. Though limited to one family, these results are a paradigmatic example of how genetic variants may represent useful treatment markers, able to aid clinicians in predicting response and in prescribing the therapeutic approach most likely to succeed in the single patient.

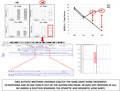

## 1 INTRODUCTION

Children with autism spectrum disorder (ASD) display impressive interindividual differences in clinical symptoms, developmental trajectories, and treatment response (Persico, Cucinotta, Ricciardello, & Turriziani, 2020). Evidence‐based early interventions targeting children between 18 months and 4 years of age, can be broadly distinguished into two classes: naturalistic developmental behavioral approaches, such as the Early Start Denver Model (ESDM), and highly structured approaches based on the principles of Applied Behaviour Analysis, namely Early Intensive Behavioral Interventions (EIBI) (Reichow, Hume, Barton, & Boyd, 2018). Marked variation in individual response to early intervention is observed in clinical practice, as well as differential efficacy of naturalistic versus structured approaches in single patients (Vivanti, Prior, Williams, & Dissanayake, 2014). Early intervention strategies are currently prescribed on the basis of local service availability and clinical experience, rather than on each child's characteristics and underlying neurobiology. Not surprisingly, their outcome is good or excellent only in 19.7% of the children, fair or poor in 31.1% and 47.7% of cases, respectively (Steinhausen, Mohr Jensen, & Lauritsen, 2016). Objective biomarkers could help clinicians prescribe targeted treatments, fostering earlier and more effective interventions, while promoting an efficient use of available clinical resources.

Genetics strongly contributes to ASD, with the majority of cases displaying highly heterogeneous gene x gene interactions (Fernandez & Scherer, 2017). Genetic variants modulate different features of the disorder and could influence also responsiveness to behavioral treatment. In this study we present the differential treatment outcome of two brothers with ASD from a single multiplex family, both initially treated applying exactly the same naturalistic ESDM approach. The presence of a maternally inherited 65 Kb deletion of chr. 13q32.2 involving the dendritic and synaptic gene *FARP1* [OMIM n. 602654] was detected in the ESDM “non‐responding” affected sibling, who later responded at least partially to a highly structured EIBI. Instead his older brother, who does not carry the deletion, during ESDM treatment acquired expressive language and came out of the autism spectrum. The absence of other genetic abnormalities able to explain this dramatic difference in treatment outcome, supports the role of *FARP1* as a neural plasticity and “naturalistic treatment response” gene in this family.

## 2 CLINICAL REPORT

The probands are two brothers born in 2010 and 2012, respectively, from nonconsanguineous parents with a positive family history for ASD, schizophrenia, and Klinefelter syndrome. Both brothers were born by Caesarean section at 36 weeks. The first‐born's (FB) birth weight was 2,970 g, perinatal history was uneventful, and psychomotor development was typical until 12 months of life, when parents observed the presence of autistic socio‐communicative deficits. The second‐born's (SB) birth weight was 1,940 g (his C‐section was programmed due to fetal growth delay). At birth, Apgar score was 9 at 1 min and he presented with hypoglycemia and two congenital ventricular septal defects, which resolved spontaneously in subsequent months. Pyloromyotomy for congenital hypertrophic pyloric stenosis was performed at 2 months of age. His psychomotor development was characterized by autonomous walking at 20 months preceded by slow and asymmetrical crawling, while babbling began at 26 months. For the two brothers, a diagnosis of ASD was established at the age of 17 and 18 months, respectively. Hearing and visual sensory deficits, motor impairment, traumatic brain injury, other genetic disorders associated with ASD (e.g., Fragile X syndrome), seizures, and prenatal drug exposure were excluded for both brothers. EEG and MRI were negative.

The diagnostic assessment, performed at the “G. Martino” University Hospital between the years 2012 and 2017, is described in the Supplementary Methods. At baseline (T0), superimposable scores were recorded for FB and SB using the Autism Diagnostic Observation Schedule‐2 (ADOS‐2) and the Griffiths Mental Development Scales (GMDS) (Figures S1 and S2). Both brothers were reassessed after 6 (T1) and 12 months (T2) of ESDM treatment, as well as following long post‐treatment time intervals, i.e. at 7 and 5 years old for FB and SB, respectively (T3).

Early Start Denver Model treatment, a comprehensive and naturalistic behavioral intervention for infants with ASD (Rogers & Dawson, 2010), was performed for 12 months at the Interdepartmental Program "Autism 0‐90", during 90‐min daily sessions for 4 days/week. The same three experienced therapists, periodically supervised by Dr. Costanza Colombi (Univ. of Michigan), were involved at two‐years distance in the treatment of both children, which was carried out in the same environment. Therapists, clinicians and parents were not aware of the genetic results at the time of behavioral intervention.

**Figure 1:**
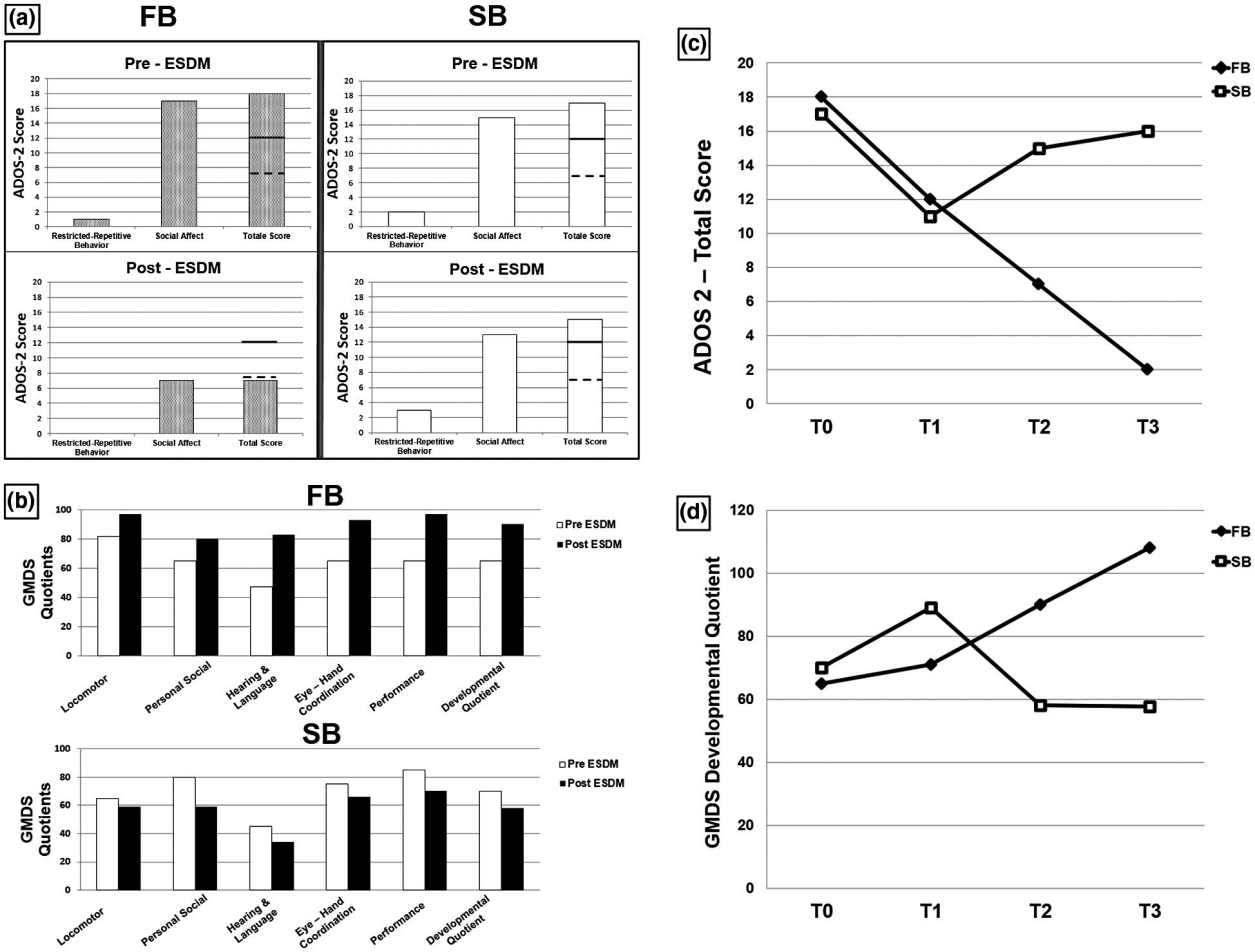
(a) ADOS‐2 scores before and after ESDM treatment for FB (left panels) and SB (right panels). 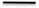 Threshold score for autism 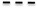 Threshold score for autism spectrum. (b) Pre‐ and Post‐treatment GMDS quotients for FB (top panel) and SB (bottom panel). Normal reference quotient: 100 ± 20. (c) Total ADOS‐2 scores at T0, T1 and T2 of ESDM treatment, and (T3) post‐treatment at the age of 7 and 5 y.o. for FB and SB, respectively. (d) GMDS Developmental Quotient at T0, T1 and T2 of ESDM treatment, and (T3) post‐treatment at the age of 7 and 5 y.o. for FB and SB, respectively. Normal reference quotient: 100 ± 20. ADOS‐2, Autism Diagnostic Observation Schedule‐2; ESDM, Early Start Denver Model; GMDS, Griffiths Mental Development Scales

Despite appearing more severely autistic than SB at intake, during ESDM treatment FB displayed prominent improvements in autism core symptoms (Figure 1a) and in all areas of neurodevelopment (Figure 1b). He no longer met the criteria for an ASD diagnosis at T2 according to two expert clinicians. His autism scores and developmental quotients improved at T2 and even further at T3, when FB was 7 years old (Figure 1c,d). Conversely, SB did not display any measurable benefit on autism core symptoms and developmental quotients from 12 months of the same ESDM intervention (Figure 1a–d) and still fully satisfied DSM‐5 diagnostic criteria for ASD at the end of treatment. SB was subsequently treated for 12 months with a highly structured EIBI (1‐hr daily sessions for 4 days/week), paired with Augmentative Alternative Communication using the Picture Exchange Communication System (PECS), during two weekly 1‐hr sessions. This approach soon yielded a partial, though still modest, clinical response, independently observed by two child neuropsychiatrists, parents, teachers and therapists, especially in terms of expressive language development (onset of circumstantial use of single words followed by progressive growth of single‐word vocabulary) and cognition (stabilization of the GMDS developmental quotient between T2 and T3 in Figure 1d), more than in core autism symptoms (see ADOS scores in Figure 1c).

## 3 GENETIC RESULTS

**Figure 2:**
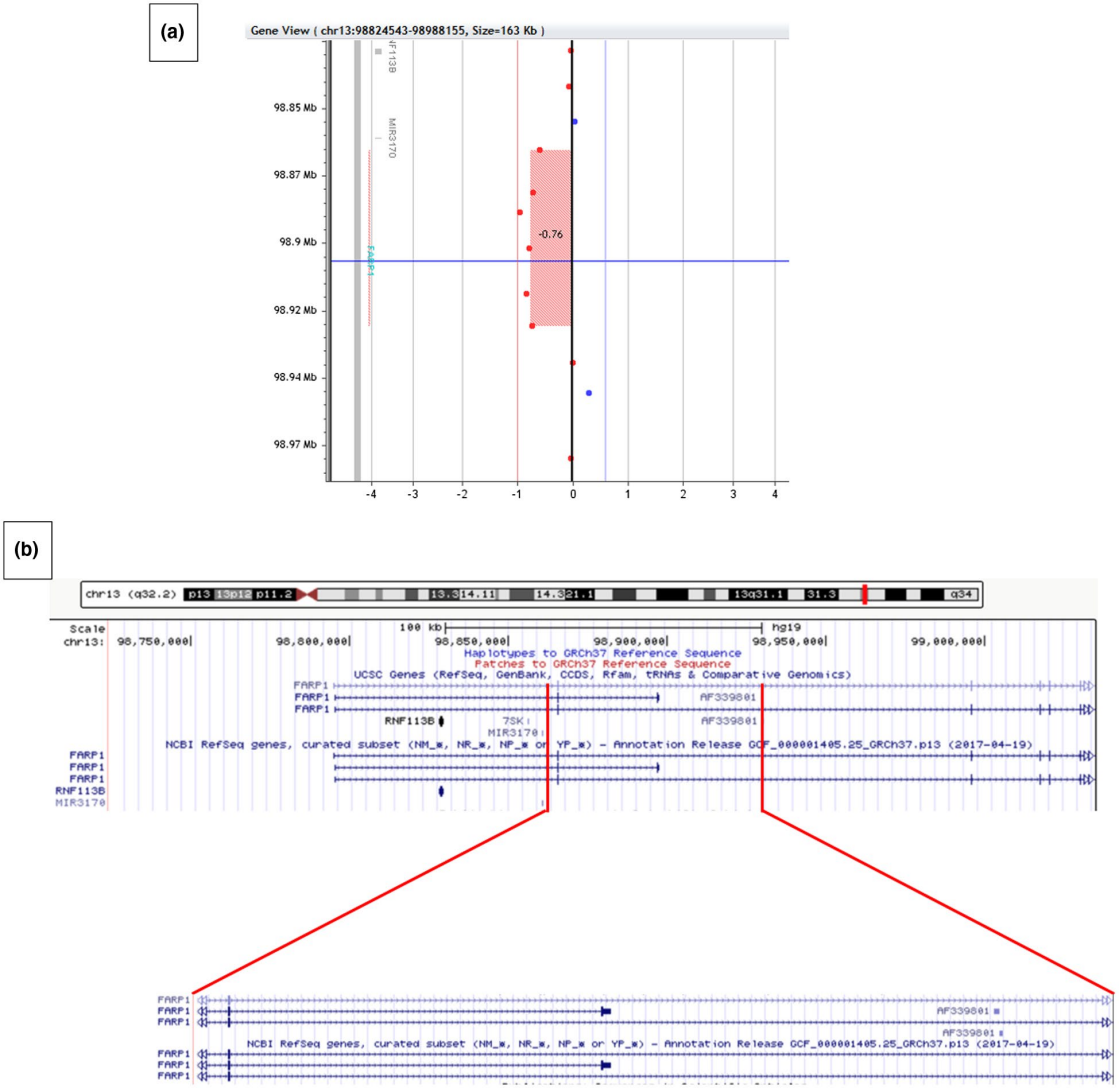
(a) Array‐CGH profile of SB at chr.13q32.2, highlighting the 65.4 kb deletion (98,865,449–98,930,894/hg19). (b) The region spanning exons 1–6 of FARP1 (top) and the deleted region encompassing exon 2, shared by all isoforms, and exon 3 of the short isoform (bottom)

DNA samples were prepared according to standard procedures after the parents gave written informed consent. Array‐CGH (Agilent 180K) and WGS (Illumina HiSeq) were performed as described in the Supplementary Methods. Array‐CGH was negative in FB. Instead, SB carries a rare maternally inherited 65 Kb deletion located in chr. 13q32.2 (98,865,449–98,930,894/hg19), involving the dendritic and synaptic gene *FARP1* (OMIM ID: 602654). Specifically, the centromeric breakpoint is just upstream of the highly conserved exon 2 and the deletion also spans exon 3 of the short isoform of *FARP1* (Figure 2, panels A and B). This deletion is a novel rare variant present neither in the DGV, nor in the Decipher database (https://decipher.sanger.ac.uk/), which lists 31 deletions or duplication (*N* = 17 and 14, respectively) encompassing *FARP1*, but all much larger (1.43–95.66 Mb) than the deletion carried by SB. WGS confirmed the same rare 13q32.2 deletion previously identified by a‐CGH (WGS hg19 coordinates—chr 13:98,862,822–98,939,679). No other clinically relevant genetic variant was detected.

Collectively, the only genetic variant identified by both a‐CGH and WGS seemingly able to explain the lack of response to ESDM treatment recorded in SB is the 13q32.2 deletion, partly spanning the coding sequence of *FARP1*.

## 4 DISCUSSION

This study describes a multiplex family with two children, both diagnosed with ASD at 1½ years of age and both treated with the same early naturalistic intervention based on the ESDM. Treatment resulted in two opposite outcomes: FB, a full “responder”, was no longer autistic at the end of ESDM treatment, whereas SB was a “non‐responder”, who subsequently showed a more satisfactory, though still modest, clinical response to a structured EIBI intervention. Both children underwent the exact same intervention applied by the same therapists. Hence this dramatic difference in treatment outcome must have a strong neurobiological basis.

The only genetic variant seemingly able to explain the observed differences in ESDM treatment response between the two siblings, as detected both by a‐CGH and WGS, is the 13q32.2 deletion, partly spanning the coding sequence of *FARP1*. *FARP1* (FERM, Rho/ArhGEF and Pleckstrin domain protein1) encodes for a postsynaptic multi‐domain protein that interacts with cell surface proteins, regulating synapse function and morphology, and with the intracellular GTPase Rac1 to promote actin assembly, dendritic growth and synaptogenesis (Figure S3). The cell‐type specific functions of Farp1 are profoundly affected by its subcellular distribution and cell surface binding partners. These include:

1. SynCAM1, promoting an increase in excitatory synapses and their stabilization, while modulating the composition of presynaptic active zones, ultimately fostering long‐term plasticity (Biederer et al., 2002; Cheadle & Biederer, 2014; Fogel et al., 2007; Robbins et al., 2010). Loss of SynCAM1 is associated with impaired social behavior in mouse (Fujita et al., 2008), and two missense mutations in this gene have been found associated with ASD (Zhiling et al., 2008);
2. Plexins‐A4 and –A6, involved in axon guidance and extension, as well as in dendritic growth in spinal motor neurons (Cheadle & Biederer, 2012; Zhuang, Su, & Sockanathan, 2009).
3. The Neuropilin1/PlexinA1 receptor complex, responsible for recruiting Farp1 to dendrites in CA1 hippocampal neurons, where it acts downstream of soluble Sema3A to promote dendritic complexity and spine number, both in activity‐dependent and –independent manner (Cheadle & Biederer, 2014).

Most available data support Farp1 involvement in neuronal plasticity and learning. By regulating dendritic and synaptic architecture, Farp1 may thus play a pivotal role in promoting the neuroplastic events underlying clinical response to treatment.

Additional differences between the two siblings lend further support to the clinical relevance of this deletion: (a) SB was diagnosed with two congenital ventricular septal defects, which resolved spontaneously. Atrioventricular (AV) cushion development is stimulated by SEMA6D‐triggered Rho activation through PlexinA1 and Farp1 in mouse (Peng et al., 2016); (b) although there is no direct evidence linking *FARP1* to congenital pyloric stenosis, which required SB to undergo pyloromyotomy at 2 months of life, this gene is indeed expressed in the intestine and stomach wall (Koyano et al., 1997); (c) additional neurodevelopmental components are present only in SB and not in FB’s history, including delayed acquisition of autonomous walking and profoundly delayed expressive language onset with major phonological distorsions. Collectively, these broader phenotypic differences between the two siblings lend further support to *FARP1* hemizygosity as possibly relevant both to hampered brain neuroplasticity and to peripheral organs, such as the heart.

This report, while limited to a single family, *is unique in many ways because all variables potentially able to introduce a methodological bias in treatment studies were controlled*. The present results are a paradigmatic example of how genetic variants may aid clinicians in prescribing the therapeutic approach most likely to succeed in the single patient. More broadly, gene networks and functional pathways predisposing to optimal naturalistic versus structured treatment response may be ultimately more useful than single rare genetic variants, each likely carried by a very small number of affected children (Parikshak, Gandal, & Geschwind, 2015). Genes like *FARP1* may represent plausible candidates to influence neuroplastic responses to naturalistic environmental stimulation and, consequently, preferential intervention strategy in ASD or even transdiagnostically. Although WGS has not detected any other genetic variant likely to explain the different phenotypes in these two brothers, we cannot exclude that epigenetic variants may have played a role, since monozygotic twins discordant for an ASD diagnosis have been shown to differ in DNA methylation patterns, including at loci relevant to neurodevelopment (Wong et al., 2014). Future research will have to identify and follow‐up more children with deletions or mutations involving *FARP1*, to examine their developmental trajectories and behavioral treatment response. More broadly, it will be critical to establish whether specific genetic markers and/or gene networks are endowed with sufficient robustness to be applicable in the clinics to small children diagnosed with ASD and to high‐risk infant siblings (D’Abate et al., 2019).

## CONFLICT OF INTEREST

The authors declare no conflict of interest.

## AUTHORS’ CONTRIBUTIONS

A.M.P., T.B., and S.W.S. conceived the study and participated in the study design; F.C., A.R., L.T., M.Bri., and A.M.P. collected the patients’ history, performed the medical work‐up and collected blood samples; M.Bo., F.B. and G.C. performed psychological testing and questionnaire scoring; F.L.F.B., S.M., M.Bru., and T.D.B. performed the ESDM therapy under the supervision of C.C.; S.M. applied the EIBI, and M.B. performed the AAC; M.Ba., C.P., and P.C. performed the genomic DNA extraction, a‐CGH and CNV validation; P.T., C.L. and R.S. analyzed a‐CGH and neuropsychological testing data; B.K. performed W.G.S.; A.M.P. and F.C. wrote the manuscript; T.B. and S.W.S. revised the manuscript. All authors approved the final manuscript as submitted and agree to be accountable for all aspects of the work.

## Supporting information

Supplementary Material

Fig S1

Fig S2

Fig S3

## Data Availability

WGS data are accessible to researchers by applying to the MSSNG database (https://research.mss.ng/).

## References

[mgg31373-bib-0001] Biederer, T. , Sara, Y. , Mozhayeva, M. , Atasoy, D. , Liu, X. , Kavalali, E. T. , & Südhof, T. C. (2002). SynCAM, a synaptic adhesion molecule that drives synapse assembly. Science, 297, 1525–1531. 10.1126/science.1072356 12202822

[mgg31373-bib-0002] Cheadle, L. , & Biederer, T. (2012). The novel synaptogenic protein Farp1 links postsynaptic cytoskeletal dynamics and transsynaptic organization. The Journal of Cell Biology, 199, 985–1001. 10.1083/jcb.201205041 23209303 PMC3518221

[mgg31373-bib-0003] Cheadle, L. , & Biederer, T. (2014). Activity‐dependent regulation of dendritic complexity by semaphorin 3A through Farp1. The Journal of Neuroscience, 34, 7999–8009. 10.1523/JNEUROSCI.3950-13.2014 24899721 PMC4044256

[mgg31373-bib-0004] D'Abate, L. , Walker, S. , Yuen, R. K. C. , Tammimies, K. , Buchanan, J. A. , … Scherer, S. W. (2019). Predictive impact of rare genomic copy number variations in siblings of individuals with autism spectrum disorders. Nature Communications, 10, 5519 10.1038/s41467-019-13380-2 PMC6892938 31801954

[mgg31373-bib-0005] Fernandez, B. A. , & Scherer, S. W. (2017). Syndromic autism spectrum disorders: Moving from a clinically defined to a molecularly defined approach. Dialogues in Clinical Neuroscience, 19, 353–371. 29398931 10.31887/DCNS.2017.19.4/sscherer PMC5789213

[mgg31373-bib-0006] Fogel, A. I. , Akins, M. R. , Krupp, A. J. , Stagi, M. , Stein, V. , & Biederer, T. (2007). SynCAMs organize synapses through heterophilic adhesion. The Journal of Neuroscience, 27, 12516–12530. 10.1073/pnas.0712298105 18003830 PMC6673342

[mgg31373-bib-0007] Fujita, E. , Tanabe, Y. , Shiota, A. , Ueda, M. , Suwa, K. , Momoi, M. Y. , & Momoi, T. (2008). Ultrasonic vocalization impairment of Foxp2 (R552H) knockin mice related to speech‐language disorder and abnormality of Purkinje cells. Proceedings of the National Academy of Sciences of the United States of America, 105, 3117–3122. 10.1073/pnas.0712298105 18287060 PMC2268594

[mgg31373-bib-0008] Koyano, Y. , Kawamoto, T. , Shen, M. , Yan, W. , Noshiro, M. , Fujii, K. , & Kato, Y. (1997). Molecular cloning and characterization of CDEP, a novel human protein containing the ezrin‐like domain of the band 4.1 superfamily and the Dbl homology domain of Rho guanine nucleotide exchange factors. Biochemical and Biophysical Research Communications, 241, 369–375. 10.1006/bbrc.1997.7826 9425278

[mgg31373-bib-0009] Parikshak, N. N. , Gandal, M. J. , & Geschwind, D. H. (2015). Systems biology and gene networks in neurodevelopmental and neurodegenerative disorders. Nature Reviews Genetics, 16, 441–458. 10.1038/nrg3934 PMC4699316 26149713

[mgg31373-bib-0010] Peng, Y. , Song, L. , Li, D. , Kesterson, R. , Wang, J. , Wang, L. , … Jiao, K. (2016). Sema6D acts downstream of bone morphogenetic protein signaling to promote atrioventricular cushion development in mice. Cardiovascular Research, 112, 532–542. 10.1093/cvr/cvw200 28172500 PMC5901116

[mgg31373-bib-0011] Persico, A. M. , Cucinotta, F. , Ricciardello, A. , & Turriziani, L. Autisms In Rubenstein J. L. R., Rakic P., Chen B. Kwan K. & Wynshaw‐Boris A. (Eds.), Neurodevelopmental disorders (1st ed., pp. 35–77). Comprehensive developmental neuroscience London, UK: Academic Press (Elsevier Inc); 2020. ISBN‐978‐0‐12‐814409‐1.

[mgg31373-bib-0012] Reichow, B. , Hume, K. , Barton, E. E. , & Boyd, B. A. (2018). Early intensive behavioural intervention (EIBI) for young children with autism spectrum disorders (ASD). Cochrane Database of Systematic Reviews, 5, CD009260 10.1002/14651858.CD009260.pub3 29742275 PMC6494600

[mgg31373-bib-0013] Robbins, E. M. , Krupp, A. J. , Perez De Arce, K. , Ghosh, A. K. , Fogel, A. I. , Boucard, A. , … Biederer, T. (2010). SynCAM 1 adhesion dynamically regulates synapse number and impacts plasticity and learning. Neuron, 68, 894–906. 10.1016/j.neuron.2010.11.003 21145003 PMC3026433

[mgg31373-bib-0014] Rogers, S. J. , & Dawson, G. (2010). Early Start Denver Model for young children with autism. New York, NY: Guilford Press ISBN ‐978‐1606236321. https://www.guilford.com/books/Early‐Start‐Denver‐Model‐Young‐Children‐Autism/Rogers‐Dawson/9781606236314/reviews.

[mgg31373-bib-0015] Steinhausen, H. C. , Mohr Jensen, C. , & Lauritsen, M. B. (2016). A systematic review and meta‐analysis of the long‐term overall outcome of autism spectrum disorders in adolescence and adulthood. Acta Psychiatrica Scandinavica, 133, 445–452. 10.1111/acps.12559 26763353

[mgg31373-bib-0016] Vivanti, G. , Prior, M. , Williams, K. , & Dissanayake, C. (2014). Predictors of outcomes in autism early intervention: Why don't we know more? Frontiers in Pediatrics, 2, 58 10.3389/fped.2014.00058 24999470 PMC4064565

[mgg31373-bib-0017] Wong, C. C. , Meaburn, E. L. , Ronald, A. , Price, T. S. , Jeffries, A. R. , Schalkwyk, L. C. , … Mill, J. (2014). Methylomic analysis of monozygotic twins discordant for autism spectrum disorder and related behavioural traits. Molecular Psychiatry, 19(4), 495–503. 10.1038/mp.2013.41 23608919 PMC3906213

[mgg31373-bib-0018] Zhiling, Y. , Fujita, E. , Tanabe, Y. , Yamagata, T. , Momoi, T. , & Momoi, M. Y. (2008). Mutations in the gene encoding CADM1 are associated with autism spectrum disorder. Biochemical and Biophysical Research Communications, 377, 926–929. 10.1016/j.bbrc.2008.10.107 18957284

[mgg31373-bib-0019] Zhuang, B. , Su, Y. S. , & Sockanathan, S. (2009). FARP1 promotes the dendritic growth of spinal motor neuron subtypes through transmembrane Semaphorin6A and PlexinA4 signaling. Neuron, 61, 359–372. 10.1016/j.neuron.2008.12.022 19217374 PMC2654783

