# Supplementary material for: FARP‐1 deletion is associated with lack of response to autism treatment by early start denver model in a multiplex family
Source: Mol Genet Genomic Med. 2020 Jun 25;8(9):e1373. doi: 10.1002/mgg3.1373 (PMC7507005; doi:10.1002/mgg3.1373)
Supplement: Supplementary file 1 — Supplementary Material [file MGG3-8-e1373-s001.pdf]

SUPPLEMENTAL INFORMATION

FARP-1 DELETION IS ASSOCIATED WITH LACK OF RESPONSE TO AUTISM TREATMENT BY EARLY START DENVER MODEL IN A MULTIPLEX FAMILY

Running title: *FARP-1* and response to autism treatment

Francesca Cucinotta[1], Arianna Ricciardello[1], Laura Turriziani[1], Calabrese Giorgia[1], Marilena Briguglio[1], Maria Boncoddo[1], Fabiana Bellomo[1], Pasquale Tomaiuolo[1], Silvia Martines[1], Marianna Bruschetta[1], Francesca La Fauci Belponer[1], Tiziana Di Bella[1], Costanza Colombi[2], Marco Baccarin[3], Chiara Picinelli[3], Paola Castronovo[3], Carla Lintas[4], Roberto Sacco[4], Thomas Biederer[5], Barbara Kellam[6,7], Stephen W. Scherer[6,7,8,9], and Antonio M. Persico[1]

(1) Interdepartmental Program "Autism 0-90", "G. Martino" University Hospital of Messina, Messina, Italy;
(2) Department of Psychiatry, University of Michigan, Ann Arbor (MI), USA;
(3) Mafalda Luce Center for Pervasive Developmental Disorders, Milan, Italy;
(4) Service for Neurodevelopmental Disorders & Laboratory of Molecular Psychiatry and Neurogenetics, University “Campus Bio-Medico”, Rome, Italy;
(5) Department of Neurology, Yale University School of Medicine, New Haven (CT), USA;
(6) Genetics & Genome Biology Program, and
(7) The Centre for Applied Genomics, The Hospital for Sick Children, Toronto, Canada;
(8) Department of Molecular Genetics, University of Toronto, Toronto, Canada;
(9) McLaughlin Centre, University of Toronto, Toronto, Canada.

Corresponding Author: Prof. Antonio Persico, Interdepartmental Program "Autism 0-90", "G. Martino" University Hospital, via Consolare Valeria 1, I-98125 Messina, Italy; phone number +39-90-2217121; email apersico@unime.it.

Supplementary Methods

Diagnostic Assessment: Both brothers initially satisfied DSM-IV-TR diagnostic criteria for Autistic Disorder (1) and DSM-5 criteria for ASD (2), based on direct assessment by two expert child neuropsychiatrists. The clinical diagnosis was confirmed using the Autism Diagnostic Observation Schedule (ADOS-G) (3) for FB, and the Autism Diagnostic Observation Schedule-2 (ADOS-2) (4) for SB. ADOS-G scores for Social Affect (SA) and Restrictive and Repetitive Behaviors (RRB) were converted into ADOS-2 scores to allow comparisons for each subscale. Developmental level was measured using the Griffiths Mental Development Scales (GMDS) (5). Following baseline assessment (T0), both brothers underwent ESDM treatment for 12 months (see below) and were re-assessed at mid-term treatment (T1 = 6 months), at the end of treatment (T2=12 months) and after long post-treatment time intervals (T3 = at the age of 7 and 5 y.o. for FB and SB, respectively). After 12 months of ESDM intervention, FB displayed prominent improvements in autism core symptoms (Fig.1A, left panels) and in all areas of neurodevelopment (Fig. 1B, top). He no longer met criteria for an ASD diagnosis at T2, according to two expert clinicians. Autistic symptoms and developmental quotients improved over the entire duration of the 12-month treatment period and even further at T3, when FB was 7 y.o. (Figs. 1C and 1D). Conversely, SB did not display any measurable benefit on autism core symptoms from 12 months of the same ESDM intervention (Fig. 1A, right panels) and still fully satisfied DSM-5 diagnostic criteria for ASD at the end of treatment. Similarly, standardized GMDS quotients recorded no sizable improvement in global neurodevelopment (Fig. 1B, bottom panel). Interestingly, SB displayed an initial improvement at T1, overlapping with the positive trend displayed by FB, but this response was lost between T1 and T2 (Figs. 1C and 1D).

Array-CGH. Both parents gave written informed consent to the full panel of genetic assessments, as approved by the IRB of Univ. of Messina, and to the publication of the present report. Blood was drawn from all family members, DNA was extracted and a-CGH analysis was performed at the Mafalda Luce Center for Pervasive Developmental Disorders (Milan, Italy), as previously described (6). Briefly, the Human Genome CGH SurePrint G3 Microarray 4 x 180K Kit (Agilent Technologies, Santa Clara, CA) was employed and quality control was performed using the Agilent Feature Extraction v10.7. CNV call was performed using the ADM-2 algorithm, as implemented in the Agilent Cytogenomic Software v2.9, and

CNVs were annotated also in reference to the gold standard variants in the Database of Genomic Variants (DGV) (7,8). Duplications and deletions were defined based on a minimum of 3 consecutive probes. *De novo* CNVs and potentially relevant inherited CNVs with ambiguous Log_2 Ratio profiles were validated by RT-PCR using TaqMan assays, whenever available, or selective PCR amplification and SybrGreen. All genome coordinates in this manuscript refer to NCBI Build 37 (UCSC hg19).

<u>*Whole Genome Sequencing*</u>. WGS was performed at The Centre for Applied Genomics (Toronto, Canada) using aliquots of the same genomic DNA extracted from whole blood and analysed by a-CGH, as previously described (9,10). Briefly, sequencing was performed with the Illumina HiSeq X system and following Illumina's recommended protocols. Base calling and data analysis were performed using Illumina HiSeq Analysis Software (HAS) version 2-2.5.55.1311. Reads were mapped to the hg19 reference sequence using Isaac alignment software (Isaac alignment software: SAAC00776.15.01.27), and single nucleotide variants (SNVs) and small indel variants were detected using the Isaac variant caller [Isaac Variant Caller (Starling): 2.1.4.2]. These variants were annotated using a custom pipeline based on ANNOVAR (11). Rare variants were defined as those with ≤ 1% frequency in large public control databases (12-14). CNVs were also analysed to replicate and extend a-CGH results obtained at the Mafalda Luce Center, using the read-depth methods ERDS (15) and CNVnator (16) (applying a window size of 500 bp). High-quality CNVs were defined as those greater than 1 kb and detected by both ERDS and CNVnator with more than 50% reciprocal overlap (17). Rare CNVs are those with ≤ 1% frequency in the Autism Speaks MSSNG dataset (probands and parents) (9). CNVs were also annotated with respect to overlap with variants present in DGV (7,8) and in a control dataset comprised of 10,851 unrelated subjects, with a majority being of European ancestry, who were genotyped on multiple microarray platforms including the Affymetrix Genome-wide Human SNP Array 6.0, Illumina HumanOmni2.5, and Affymetrix CytoScan HD (18,19). Variants of interest were confirmed by PCR and Sanger sequencing or by chromosomal microarrays.

References

(1) American Psychiatric Association. Diagnostic and Statistical Manual of Mental Disorders, (4th edn.) Washington, DC: American Psychiatric Association; 2006.

(2) American Psychiatric Association. Diagnostic and Statistical Manual of Mental Disorders, (5th edn.). Arlington, VA: American Psychiatric Association; 2013.

(3) Lord C, Risi S, Lambrecht L, et al. The autism diagnostic observation schedule-generic: a standard measure of social and communication deficits associated with the spectrum of autism. J Autism Dev Disord 2000; 30:205-223.

(4) Lord C, Rutter M. *Autism Diagnostic Observation Scales - 2nd Edition* (ADOS-2). Torrance, CA: Western Psychological Services. 2012.

(5) Luiz DM, Barnard A, Knoesen N, et al. *Griffiths Mental Developmental Scales-Extended Revised: Two to Eight Years. Analysis Manual*. Oxford, UK: Hogrefe. 2006.

(6) Picinelli C, Lintas C, Piras IS, et al. Recurrent 15q11.2 BP1-BP2 microdeletions and microduplications in the etiology of neurodevelopmental disorders. Am J Med Genet B Neuropsychiatric Genet 2016; 171:1088-1098.

(7) MacDonald JR, Ziman R, Yuen RK, et al., The Database of Genomic Variants: a curated collection of structural variation in the human genome. Nucleic Acids Res 2014; 42:D986-992.

(8) Zarrei M, MacDonald JR, Merico D, Scherer SW. A copy number variation map of the human genome. Nature Rev Genet 2015; 16:172-183.

(9) Yuen RK, Merico D, Bookman MJLH, et al. Whole genome sequencing resource identifies 18 new candidate genes for autism spectrum disorder. Nature Neurosci 2017; 20:602-611.

(10) Lionel AC, Costain G, Monfared N, et al. Improved diagnostic yield compared with targeted gene sequencing panels suggests a role for whole-genome sequencing as a first-tier genetic test. Genet Med 2018; 20:435-443.

(11) Wang K, Li M, Hakonarson H. ANNOVAR: functional annotation of genetic variants from high-throughput sequencing data. Nucleic Acids Res 2010; 38:e164.

(12) 1000 Genomes Project Consortium, Abecasis GR, Altshuler D, et al. A map of human genome variation from population-scale sequencing. *Nature* 2010; 467:1061-1073.

(13) Tennessen JA, Madeoy J, Akey JM. Signatures of positive selection apparent in a small sample of human exomes. Genome Res 2010; 20:1327-1334.

(14) Lek M, Karczewski KJ, Minikel EV, et al. Analysis of protein-coding genetic variation in 60,706 humans. Nature 2016; 536:285-291.

(15) Zhu M, Need AC, Han Y, et al. Using ERDS to infer copy-number variants in high-coverage genomes. Am J Hum Genet 2012; 91:408-421.

(16) Abyzov A, Urban AE, Snyder M, Gerstein M. CNVnator: an approach to discover, genotype, and characterize typical and atypical CNVs from family and population genome sequencing. Genome Res 2011; 21:974-984.

(17) Trost B, Walker S, Wang Z, et al. A comprehensive workflow for read depth-based identification of copy-number variation from whole-genome sequence data. Am J Hum Genet 2018; 102:142-155.

(18) Uddin M, Thiruvahindrapuram B, Walker S, et al. A high-resolution copy-number variation resource for clinical and population genetics. Genet Med 2015; 17:747-752.

(19) Uddin M, Pellecchia G, Thiruvahindrapuram B, et al. Indexing Effects of Copy Number Variation on Genes Involved in Developmental Delay. Sci Rep 2016; 6:28663.

Supplementary Figure legends

Supplementary Figure S1. Similar pre-treatment ADOS-2 scores in the two siblings, FB and SB.

Supplementary Figure S2. Similar pre-treatment GMDS quotients in the two siblings, FB and SB (normal reference score 100 $\pm$ 20).

Supplementary Figure S3. Direct acyclic graph of Gene Ontology biological process categories for FARP1.
