# Supplementary figures and images for: FARP‐1 deletion is associated with lack of response to autism treatment by early start denver model in a multiplex family
Source: Mol Genet Genomic Med. 2020 Jun 25;8(9):e1373. doi: 10.1002/mgg3.1373 (PMC7507005; doi:10.1002/mgg3.1373)

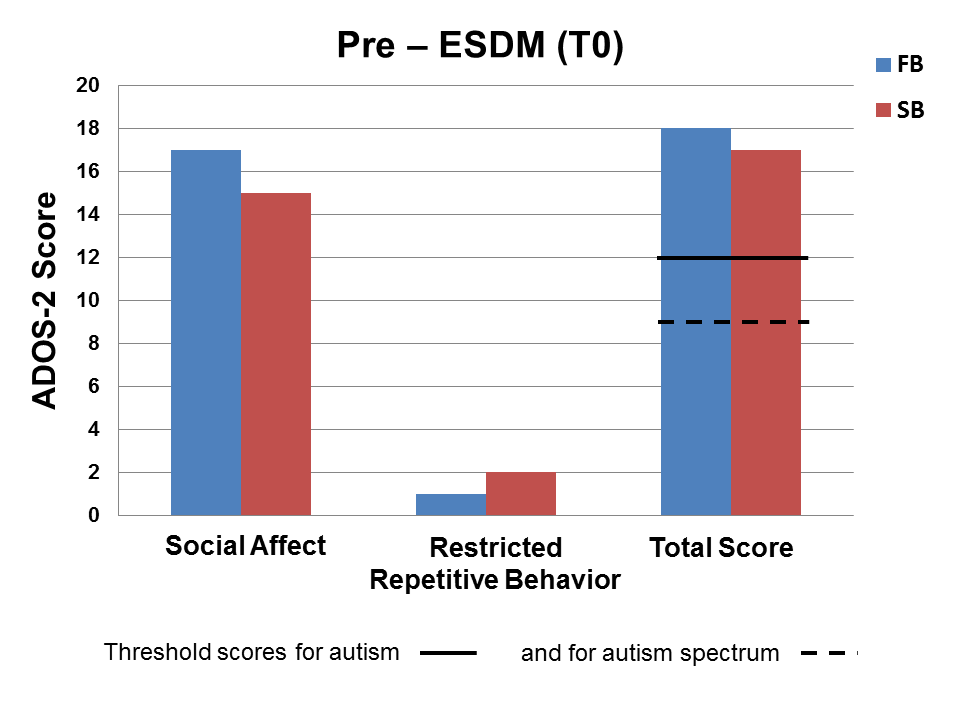

Supplement: Supplementary file 2 — Fig S1 [file MGG3-8-e1373-s002.TIF]

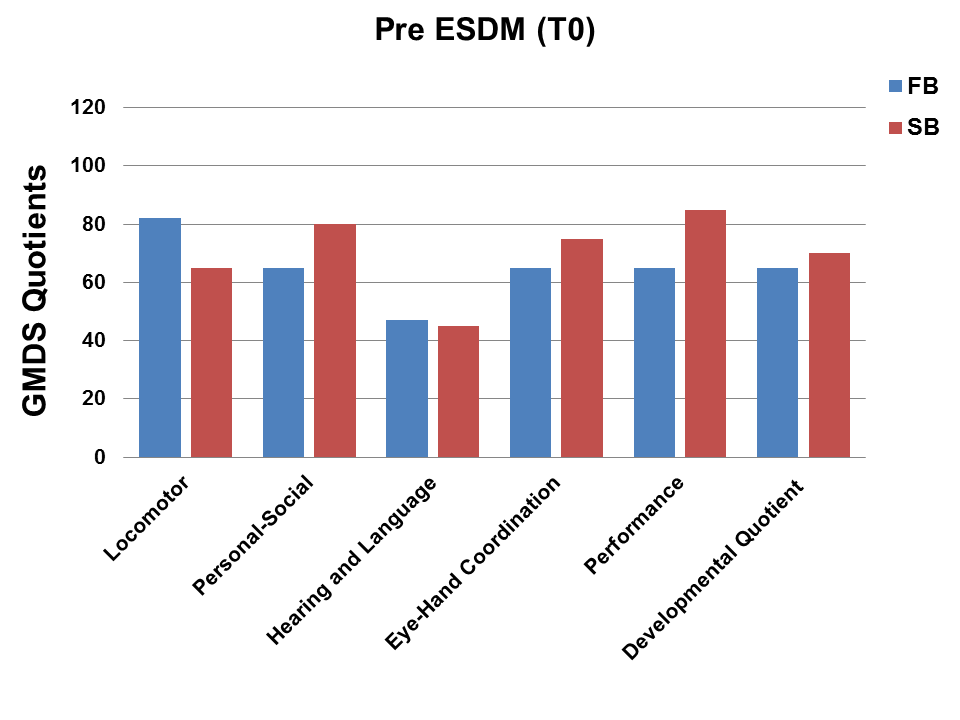

Supplement: Supplementary file 3 — Fig S2 [file MGG3-8-e1373-s003.tif]

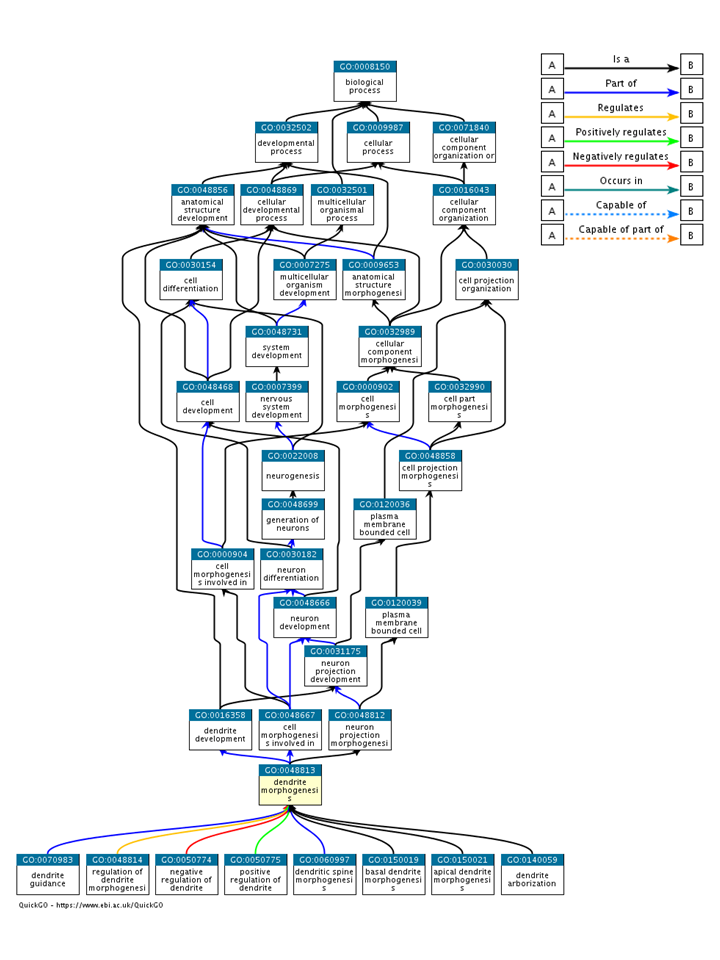

Supplement: Supplementary file 4 — Fig S3 [file MGG3-8-e1373-s004.tif]
